# Growth and Spoilage Potential of an *Aeromonas salmonicida* Strain in Refrigerated Atlantic Cod (*Gadus morhua*) Stored under Various Modified Atmospheres

**DOI:** 10.3390/foods11182757

**Published:** 2022-09-08

**Authors:** Sunniva Hoel, Jørgen Lerfall, Anita Nordeng Jakobsen

**Affiliations:** Department of Biotechnology and Food Science, NTNU—Norwegian University of Science and Technology, NO-7491 Trondheim, Norway

**Keywords:** *Aeromonas*, seafood, Atlantic cod, spoilage, modified atmosphere packaging (MAP), vacuum packaging, inoculation, trimethylamine (TMA)

## Abstract

*Aeromonas* spp. are ubiquitous aquatic bacteria, frequently isolated from seafood. The growth and spoilage potential of an inoculated strain of *Aeromonas salmonicida* (SU2) in Atlantic cod (*Gadus morhua*) fillets were assessed as a function of various modified atmospheres (MA) (CO_2_ (67%) with O_2_ (33%) or N_2_ (33%), and vacuum packaging (VP)) under refrigerated storage (4 °C) for 13 days. Additionally, the general microbiological quality was assessed by quantifying total aerobic psychrotrophic count (PC), total aerobic plate count (APC), and H_2_S-producing bacteria. A selection of spoilage metabolites was quantified by nuclear magnetic resonance (NMR) spectroscopy. The MA containing CO_2_/O_2_ and CO_2_/N_2_ significantly inhibited the growth of the inoculated strain throughout storage (*p* < 0.001), whereas VP allowed for a 3-log increase of *Aeromonas* in 13 days. No significant effect of the inoculation with *A. salmonicida* on spoilage metabolite production was detected. Applying O_2_ instead of N_2_ in the CO_2_-enriched atmosphere resulted in a significantly lower PC for 10 days, and H_2_S-producing bacteria were inhibited in both MAs compared to vacuum. This study provides data that can be used as a basis to further elucidate the role of bacteria belonging to the genus *Aeromonas* as potential spoilage organisms in marine fish species.

## 1. Introduction

*Aeromonas* spp. are ubiquitous aquatic bacteria, highly prevalent in fish and crustaceans, and commonly recognized as spoilage organisms in various types of seafood [1]. There are currently 36 recognised species of *Aeromonas* [2], of which *Aeromonas salmonicida* is one of those most frequently associated with seafood spoilage [3]. *Aeromonas* spp. can normally grow in seafood under refrigeration temperatures [4,5,6] and are in several studies recognised as a major part of the spoilage microbiota of ice-stored seafood products from tropical waters such as sea bream (*Sparus aurata*) [7], common carp (*Cyprinus carpio*) [8], sea salmon (*Pseudopercis semifasciata*) [9], and farmed shrimps (*Litopenaeus vannamei*) [10]. *Aeromonas media* was isolated from the late spoilage of silver carp (*Hypophthalmichtys molitrix*) surimi preserved at 4 °C [11]. However, data on the role of *Aeromonas* spp. as potential spoilage organisms in marine fish species are sparse. Despite being recognised as part of Atlantic salmon (*Salmo salar*) [12] and Atlantic cod (*Gadus morhua*) [13] microbiota, there is less knowledge of the ability of *Aeromonas* to grow and deteriorate processed marine fish fillets by producing spoilage-associated metabolites under various packaging atmospheres. 

The use of modified atmosphere (MA) packaging combined with cold storage extends the shelf life of most seafood products by inhibiting bacterial growth and oxidative reactions [14]. The effect of MA packaging and the obtained shelf-life extension depends on fish species, fat content, gas composition in the package (concentration of CO_2_ in the atmosphere and availability of oxygen), the ratio of gas to product volume, storage temperature, and the composition of the initial microbial community. Post-mortem quality deterioration of fish is caused by autolytic changes, chemical oxidations, and, to the largest extent, microbial activity [15]. Resulting from the metabolism of specific spoilage organisms (SSO), the production of volatile organic compounds (VOCs) is linked to the development of characteristic off-odours and off-flavours, leading to sensory rejection [16,17]. In particular, the pungent trimethylamine (TMA) is produced in MA and vacuum packaged products resulting from the reduction of trimethylamine oxide (TMAO) by bacteria capable of anaerobic respiration such as *Photobacterium* and *Shewanella* spp. [13,18,19]. Thus the application of O_2_ in the packaging atmosphere is used to inhibit the production of TMA in TMAO-rich fish species such as cod [14,20]. Atlantic cod is an important trade product for the seafood industry in the North Atlantic area, and the effect of various MA conditions on cod has been extensively studied [13,21,22,23]. Packaging using MA is reported to preserve quality better than vacuum packaging (VP) [19,24], and the reported shelf-life for cod under various MA conditions is in the range from 10 to 20 days at 0–3 °C [25,26]. *Photobacterium phosphoreum,* characterised by its efficient TMA production and high resistance to CO_2_, has been identified as SSO of chilled MA packaged Atlantic cod (>60% CO_2_) [20,27]. The application of gas mixtures with high levels of CO_2_ and O_2_ has been suggested to be beneficial for the shelf life of MA packaged cod [14,28]. However, the effect of high O_2_ concentrations on the fish microbiota during chilled storage is not well documented [13,29]. 

Compared to *P. phosphoreum* and *Shewanella* spp., the significance of *Aeromonas* spp. as a spoilage organism in chilled MA or vacuum packaged cod is far less studied. Inoculation studies have demonstrated that *Aeromonas* spp. can be associated with spoilage in vacuum packaged products such as *pre-rigor* filleted Atlantic salmon by the production of TMA, hypoxanthine, and H_2_S [30], and in cold smoked salmon by the production of TVBN [31]. Inoculation of *A. salmonicida* in shrimps under MA (50/50% CO_2_/N_2_) resulted in the production of off-odours described as butter, sour, sulphur, and amine [32]. Strains of *A. salmonicida* isolated from common carp produced off-odours characterised as cheese-like and sour, and the ability of the strain to reduce TMAO to TMA was confirmed [8]. 

In a recent study, Hansen et al. [13] identified *Aeromonas* spp. as one of the dominating bacteria after 13 days of VP in Atlantic cod fillets using next-generation sequencing (NGS). In the same study, they demonstrated that *Aeromonas* spp. can enumerate at a reduced rate in an atmosphere containing 60/40% CO_2_/N_2_, but not when 40% O_2_ was applied instead of N_2_. The increased popularity of cod as a conveniently packaged product calls for more knowledge about the shelf-life and quality following packaging. Moreover, no studies have specifically targeted *A. salmonicida* in MA or vacuum packaged Atlantic cod. The aim of the study was thus to assess the growth kinetic parameters and the spoilage potential of a strain of *A. salmonicida* in Atlantic cod packaged under high O_2_ (67% CO_2_ and 33% O_2_) and CO_2_/N_2_ (67%/33%) atmospheres stored in refrigerated temperature (4 °C) for 13 days. 

## 2. Materials and Methods

### 2.1. Raw Material and Experimental Design

A total of 12 individual whole, gutted Atlantic cod (*Gadus morhua*) (average weight 5.0 kg), were purchased from a local seafood company and transported to the laboratory in boxes filled with wet ice after harvest. The fish were skinned and filleted in-house upon arrival, divided into standardised pieces of 75 ± 2 g, and randomly distributed into six groups. The experiment was designed to study the effect of different modified atmosphere compositions (67% CO_2_ balanced with either 33% O_2_ or N_2_) on the growth kinetic parameters and spoilage metabolite production following inoculation with the strain *A. salmonicida* SU2 in fresh cod fillets stored under refrigeration (4 °C). Vacuum packaging (inoculated and non-inoculated) was used as a control. The experimental design thus resulted in six groups; non-inoculated control samples and inoculated samples subjected to three different packaging atmospheres (Table 1). The samples were stored at 4 °C for 13 days. Samples for microbiological analysis were taken on days 0, 2, 4, 6, 8, 10, and 13 after packaging (*n* = 3 at each sample point, except day 0, where *n* = 4). Headspace gas analysis of packaging atmosphere was performed on all packages before microbiological sampling. Samples for metabolite analysis were collected on days 0, 6, and 13 after packaging and kept at −80 °C until extraction and analysis. 

### 2.2. Inoculum Preparation

A volume of 0.1 mL of a frozen stock culture of *A. salmonicida* strain SU2 previously isolated from sushi [33] was thawed and transferred to a 250 mL baffled shake-flask containing 100 mL tryptone soy broth (TSB) (Oxoid, Oslo, Norway). The culture was grown overnight at 37 °C, and 1 mL of the overnight culture was transferred to a new shake-flask containing 100 mL TSB. The culture was again grown overnight at 8 °C to adapt to cold storage and then diluted in 0.9% NaCl to obtain an inoculum with cell concentration corresponding to approximately log 6 CFU/mL. An aliquot of 600 µL of the cold-adapted inoculum was added to the top surface of each piece of fish (Section 2.1) and distributed evenly on the surface using a sterile L-shaped spreader. The inoculated fish were allowed to dry for 30 min before packaging.

### 2.3. Packing and Storage Conditions

The inoculated and uninoculated pieces of cod were individually packaged in 230 mL semi-rigid crystalline polyethylene terephthalate (CPET) trays (C2125-1A, Færch Plast, Holstebro, Denmark) on top of an absorbent (Bewi, Hamarvik, Norway), using a semi-automatic tray sealing packaging machine (TL250, Webomatic, Bochum, Germany) to obtain an approximate 3:1 gas:product ratio (*v*:*w*). During the packaging process, the air was evacuated before filling the trays with the set gas mixes (Table 1) and sealed with the top film (40-µm polyethylene (PE), ethylene vinyl alcohol (EVOH), PA, and PET) (Topaz B-440 AF, Plastopil, Almere, The Netherlands). The oxygen transmission rate (OTR) was 66–78 cm^3^ × 25 mm × m^−2^ × 24 h × bar at 23 °C for the tray, and 2.5 cm^3^ × 40 mm × m^−2^ × 24 h × atm at 23 °C for the cover film. Food-grade gas (CO_2_, O_2,_ or N_2_) were mixed in a MAP Mix 9000 gas mixer (Dansensor, Ringsted, Denmark) to obtain the correct packaging atmospheres, which was confirmed by measuring five packaging dummies (packages with gas only) using an O_2_ and CO_2_ analyser (Check-mate 9900, Dansensor). Furthermore, the headspace gas composition was measured in each package at the time of microbiological sampling. The VP samples were packaged in 20-µm polyamide (PA)/70-µm PE bags (OTR 50 cm^3^ × m^−2^ × 24 h × bar at 23 °C, Star-Pack Productive, Boissy-l’Aillerie, France) using a vacuum machine (Supermax-C, Webomatic). Air was evacuated to an end pressure of 25 mbar before sealing.

### 2.4. Sampling and Bacterial Quantification

A 10 g slice of cod (cut vertically through the sample) was aseptically transferred to a sterile stomacher bag and diluted 1:10 with sterile peptone water (0.85% NaCl (Merck, Oslo, Norway) and 0.1% neutralised bacterial peptone (Oxoid)) and homogenised for 60 s using a Stomacher lab blender (IUL Masticator, Barcelona, Spain). Appropriate 10-fold serial dilutions were made in peptone water. *Aeromonas* spp. were quantified on Starch Ampicillin Agar (SAA) (composed of (g/L) peptone (10), beef extract (Difco, Bordeaux, France) (1), NaCl (5), soluble starch (Difco) (10), phenol red (Sigma-Aldrich, Oslo, Norway) (0.025), and agar (Oxoid) (15)) supplemented with 10 mg/L Ampicillin (Sigma-Aldrich), as described in NMKL (Nordic Committee on Food Analysis) method no. 150 [34]. The SAA plates were incubated at 37 °C for 24 ± 2 h. Lyngby’s Iron agar (IA) (Oxoid) supplemented with 0.04 % L-cysteine (Sigma-Aldrich) was applied to quantify total aerobic count (APC) and H_2_S-producing bacteria as white and black colonies, respectively, as described in NMKL method no. 184 [35]. The IA plates were incubated at 22 °C for 72 ± 6 h. Total psychrophilic count (PC) was determined using Long & Hammer agar (LH) (composed of (g/L) proteose peptone (Oxoid) (20), gelatin (Oxoid) (40), dipotassium phosphate (K_2_HPO_4_) (Merck) (1), NaCl (10), agar (15), and ammonium ferric (III) citrate) (Sigma-Aldrich) (0.25)) prepared according to NMKL method no. 184. The LH plates were added 1% NaCl to support the growth of *P. phosphoreum* and incubated at 15 °C for 6 days. 

### 2.5. Quantification of Metabolites by Nuclear Magnetic Resonance (NMR)

A selection of water-soluble metabolites (TMA: trimethylamine, IMP: inosine monophosphate, HxR: Inosine, and Hx: hypoxanthine) were analysed by nuclear magnetic resonance (NMR) according to the method described by Shumilina, et al. [36]. The metabolites were extracted from 5 g cod muscle using 7.5% trichloroacetic acid (TCA, Sigma-Aldrich), as described previously [36]. 1D ^1^H-NMR-spectra were acquired at 300 K using a Bruker Avance 600-MHz spectrometer equipped with a 5 mm z-gradient TXI (H/C/N) cryoprobe (Bruker, Billerica, MA, USA). The NMR analysis and subsequent spectra processing were performed at the NMR centre of the faculty of Natural Sciences at NTNU (Trondheim). Spectra were processed using the TopSpin 3.5 software (Bruker)), and Trimethylsilyl propanoic acid (TSP, Sigma-Aldrich) was used as an external standard for spectral calibration (0 ppm). The reported concentrations are based on an average integral value derived from three integrations of the same resonance signal. 

### 2.6. Statistical Analysis

The statistical analyses were performed using the software SPSS (version 28, IBM, Armonk, NY, USA). Statistical analyses were done using log-transformed microbial growth data, and all mean values are presented as mean ± standard deviation (SD). Log-transformed bacterial counts were fitted to the primary growth model of Baranyi and Roberts [37], available at www.combase.cc (accessed on 8 July 2022) for estimation of maximum growth rates (µ_max_) and lag phase duration. A Pearson correlation analysis was applied to determine the linear association between bacterial counts on different growth media and the changes in headspace concentration of CO_2_ and O_2_ during storage. A General Linear Model (GLM) with storage time, packaging atmosphere, and inoculation as fixed factors were applied for main effect analysis. A one-way analysis of variance (ANOVA) with Tukey’s pairwise comparison test was used for comparing experimental groups. The alpha level was set to 5% (*p* < 0.05).

## 3. Results and Discussion

### 3.1. Headspace Gas Composition in Packages

The headspace gas composition was measured at each sampling point for the samples packaged in trays. There was a significant drop in the CO_2_ concentration between day zero (67%) and day two (39.7 ± 3.2%) (*p* < 0.001) due to the diffusion of CO_2_ from the headspace to the product [38] as the CO_2_ is absorbed in the aqueous phase of the fish fillet [39]. During storage, there was a slight increase in the headspace CO_2_ concentration, reaching an average of 45.3 ± 4.4% on day 13.

For packages with O_2_ as the balancing gas, the initial concentration was set to 33% and increased significantly from day zero to day two (53.3 ± 2.3%) (*p* < 0.001) and was stable throughout storage (*p* > 0.05). Furthermore, a significant correlation between the absorption of CO_2_ in the product and the increase in head-space O_2_ concentration was observed for the 13 days storage period (r = 0.97, *p* < 0.001). The initial concentration of N_2_ was also set to 33%, and like O_2_ packages, there was a significant increase in the headspace N_2_ from day zero to day two (*p* < 0.001) at 60.5 ± 3.0%. As a function of storage time, the N_2_ concentration decreased steadily to a final concentration of 51.8 ± 1.8% on day 13. The headspace O_2_ in these packages ranged from 0.76 ± 0.71% on day two to 0.17 ± 0.13% on day 13.

### 3.2. Effect of Various Modified Atmospheres on the Microbial Quality of Non-Inoculated Cod 

**Figure 1:**
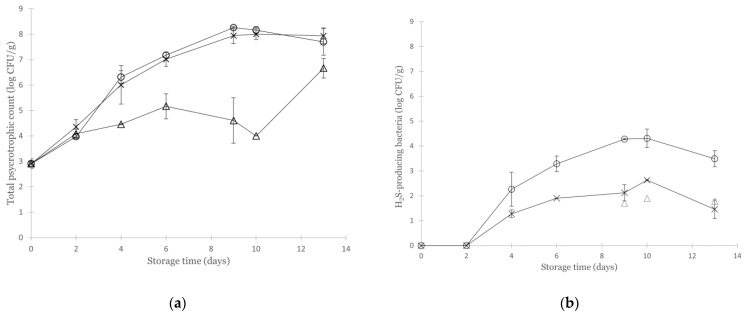
(**a**) Total aerobic psycrotrophic count (PC) quantified on Long & Hammer agar and (**b**) H_2_S-producing bacteria quantified as black colonies on Iron agar in non-inoculated cod fillets packaged in a modified atmosphere consisting of CO_2_ (67%) mixed with either O_2_ (33%) (Δ) or N_2_ (33%) (X), or in vacuum (○) for 13 days at 4 °C. *n* = 3 for each sampling point (*n* = 4 at day 0), and vertical error bars represent ± 1 SD.

The initial PC and APC in the non-inoculated samples were 2.9 ± 0.2 log CFU/g and 2.2 ± 0.2 log CFU/g, respectively, and thus within the expected level of fresh marine fish [40,41,42]. The cod were hand filleted under strict hygiene conditions, and the initial bacterial concentrations indicated a low level of contamination during processing. For the non-inoculated groups, the PC increased to 7.7–7.9 log CFU/g on day 13 for samples packaged in a vacuum and CO_2_/N_2_. Regardless of the packaging atmosphere, there was a 1 log increase in bacterial count over the first two days. However, the CO_2_/O_2_ atmosphere was the only packaging condition able to reduce bacterial growth from this point. On day 10, a significant (4-log, *p* < 0.001) difference in PC was observed between the samples packaged in CO_2_/O_2_ compared to CO_2_/N_2_ and vacuum (Figure 1a). However, this difference decreased by the end of storage, and there was no significant difference in the PC for the three non-inoculated groups at the end of storage (day 13, *p* = 0.102). The APC quantified on IA was systematically lower than the PC quantified on LH, and the difference in plate count for these two media ranged from 2.7–5.2 log CFU/g on the last sampling day. It is previously reported that the use of LH-agar gives the best quantitative determination of the total number of marine bacteria in seafood [43], which was also supported by Kuuliala et al. [21], observing higher counts on LH than IA for cod fillets due to the dominance of *Photobacterium*. IA is, however, applied for detecting bacteria able to produce H_2_S from cysteine, not exclusively *Shewanella*, but also *Aeromonas*, members of the families Enterobacteriaceae and Vibrionaceae, and some genera of lactic acid bacteria (LAB) [35]. The non-specific detection of H_2_S-producing bacteria on IA calls for using multiple indicators in the quality assessment of seafood. In the present study, no H_2_S-producing bacteria were detected in any non-inoculated sample on days 0 and 2. For samples packaged in MA containing CO_2_/O_2_, H_2_S-producing bacteria were inhibited and only detected sporadically (in 1 out of 3 parallels) at the last three sampling points. The highest increase in H_2_S-producing bacteria was observed in VP samples (maximum concentration of 4.3 log CFU/g at day 9), whereas the MA containing CO_2_/N_2_ was able to restrain the growth of H_2_S-producing bacteria to a maximum concentration of 2.6 log CFU/g (Figure 1b), which agrees with others [21,26,40,41]. Furthermore, Hansen et al. [13] demonstrated that high-oxygen packaging (60/40% CO_2_/O_2_) could inhibit common spoilage bacteria such as *Photobacterium*, *Shewanella*, and *Pseudomonas* in cod, and at the same time allow the growth of LAB (Carnobacterium) and Acinetobacter resulting in reduced sensory quality compared to products with high levels of *Photobacterium*. Another concern when applying packaging in high-oxygen atmospheres is lipid oxidation [44]; however, this is most likely not a problem in cod fillets due to the low lipid content [13].

There is no absolute association between the microbiological load and spoilage of a fish product. A limit of log 7 CFU/g has generally been considered as maximum acceptable for fish [19]; however, sensory rejection has been found at bacterial counts between 6 to 9 log CFU/g [10,21,45,46]. In MA packaged cod, Kuuliala et al. [21] found sensory rejection to occur at PC levels of 7–7.5 log CFU/g. Accordingly, the cod packaged in an atmosphere with O_2_ was the only group that never exceeded the 7 log CFU/g limit during 13 days of storage (although the PC was not significantly different from the other atmospheres at this point), and none of the groups exceeded 5 log CFU/g of H_2_S producing bacteria. No sensory assessment was done in the present study, and we cannot conclude if the products were sensory acceptable at the end of storage.

### 3.3. Growth of Aeromonas Salmonicida in Cod under Various Modified Atmospheres

**Figure 2:**
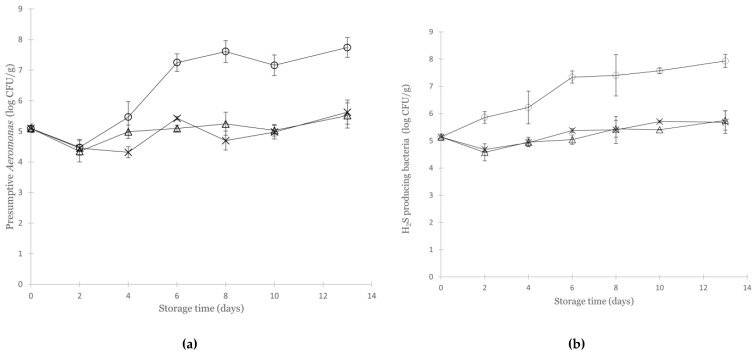
(**a**) Growth of the inoculated strain *A. salmonicida* SU2 (quantified on Starch Ampicillin Agar (SAA)) and (**b**) H_2_S-producing bacteria (black colonies on iron agar) in inoculated cod fillets packaged in CO_2_ (67%) mixed with O_2_ (33%) (Δ), N_2_ (33%) (X), or in vacuum (○) for 13 days at 4 °C. *n* = 3 for each sampling point (*n* = 4 at day 0) and vertical error bars represent ± 1 SD.

No *Aeromonas* spp. were detected in the non-inoculated control samples, and it is thus reasonable to assume that all *Aeromonas* spp. detected throughout the storage on the selective agar were the inoculated strain (*A. salmonicida* SU2). The initial concentration of the inoculated strain quantified on SAA averaged log 5.1 ± 0.1 CFU/g. The relatively high concentration of inoculum was applied to increase the likelihood of detection of metabolites in a naturally contaminated system, as previously described in Jakobsen et al. [30] and Lerfall et al. [42]. The combination of MA (balanced with either O_2_ or N_2_) and refrigeration (4 °C) inhibited the growth of *A. salmonicida* in the cod fillet during 13 days of storage (Figure 2a). There was no significant difference between the concentration of *Aeromonas* on days 0 and 13 for these two atmospheres (*p* > 0.05 for both atmospheres), and there was no significant difference in the maximum population density of *Aeromonas* under these two atmospheres: 5.5 ± 0.41 (O_2_) and 5.6 ± 0.39 (N_2_) log CFU/g, respectively (*p* > 0.05). Vacuum packaging allowed the growth of the inoculated strain, and the concentration of *Aeromonas* increased by almost 3 log units to 7.7 ± 0.32 log CFU/g during storage. The lag phase (3.4 ± 0.66 days), estimated by fitting the data to the primary growth model of Baranyi and Roberts [37], was longer than observed for the same strain in salmon under comparable conditions [30]. The maximum growth rate of the inoculated *A. salmonicida* SU strain quantified on SAA in the present study was almost two times higher in cod (µ_max_ = 1.00 ± 0.38 log CFU/day) compared to the rate previously reported in salmon (µ_max_ = 0.56 ± 0.04 log CFU/g/day) [30]. However, when comparing the growth curves for the inoculated strain quantified on SAA to the curve derived from IA, it was apparent that the inoculated culture was somewhat inhibited during the first two days, possibly from selective conditions in the SAA, resulting in a steeper slope from day two and onwards (Figure 2a).

The initial concentration of H_2_S-producing bacteria in the inoculated samples, quantified on IA, was 5.1 ± 0.1 log CFU/g. Due to the strong correlation of bacterial counts on SAA and IA (r = 0.91, *p* < 0.01), it is presumed that the black colonies counted on IA were the inoculated *A. salmonicida* strain. Taking this into consideration, the lag phase (no lag) and growth rate representing the H_2_S-producing bacteria quantified on IA (µ_max_ = 0.32 ± 0.05 log CFU/g/day) are probably more representative of the growth of *A. salmonicida* SU2 in the VP cod (Figure 2b). Despite being able to grow at different rates in refrigerated MA or VP seafood, *Aeromonas* has, to our knowledge, only once been associated with the microbiota of packaged and stored Atlantic cod [13]. Thanks to the development and increased application of NGS methods, in-depth information is being obtained about the dynamics of bacterial populations during storage and spoilage [1]. As highlighted by Parlapani et al. [7], and in the recent review by Anagnostopoulos et al. [47], the combination of culture-independent and classical microbiological methodologies must be applied to ensure the detection of bacteria that tend to escape cultivation methods, such as *Aeromonas* spp. which are likely to be overlooked on IA, LH, or on supplemented *Pseudomonas* agar (CFC) [48]. The lack of selective media for *Shewanella* and *Photobacterium* and the non-efficient selective media for *Aeromonas* and *Pseudomonas* illustrate the challenges of using only culture-dependent methods for examining the spoilage microbiota [7]. Molecular methods, in particular amplicon sequencing of the bacterial 16S rRNA gene for monitoring the most abundant and dominant microbiota of different seafood types, have revealed that *Aeromonas* are in fact more prevalent in shellfish and finfish from both cold and temperate water than previously reported by culture-dependent studies [47].

### 3.4. Spoilage Metabolite Production under Various Modified Atmospheres

Post-mortem deterioration and quality loss of seafood can be monitored by the analysis of adenosine triphosphate (ATP) degradation products as endogenous and bacterial enzymes catalyse degradation of ATP in the fish muscle through the intermediate products adenosine diphosphate (ADP), adenosine monophosphate (AMP), inosine monophosphate (IMP), Inosine (HxR), and hypoxanthine (Hx) [49]. The desired and pleasant umami taste of seafood is associated with IMP, and the further degradation of HxR and Hx by microbial enzymes is connected to the loss of freshness and the development of unpleasant sensory properties in some fish species [49,50]. 

In the present study, the concentration of IMP was significantly affected by storage time (GLM, *p* < 0.001) but not by packaging atmosphere (GLM, *p* = 0.734) or inoculation (GLM, *p* = 0.877) due to only sporadic detection during storage, which indicates that conversion of IMP to HxR had already occurred (Table 2). Overall, a significant effect of storage time and packaging atmosphere on the concentration of HxR was observed (GLM, *p* < 0.001 for both factors), but no effect following inoculation with the *Aeromonas* strain (GLM, *p* = 0.837). Regardless of inoculation, the conversion of HxR to Hx was significantly faster for samples packaged in CO_2_/N_2_ and vacuum compared to packaging in CO_2_/O_2_ (*p* < 0.05) (Table 2). Accordingly, samples depleted of HxR showed a significant increase in the Hx concentration (*p* < 0.001). A significant effect of storage time and packaging atmosphere was observed for Hx (GLM, *p* < 0.001 for both factors). In contrast, there was no significant difference in the Hx concentration in inoculated versus non-inoculated samples (GLM, *p* = 0.821). The concentration of Hx increased as a function of storage time for all groups (*p* < 0.05), and at the end of storage, the Hx concentration was significantly lower in the CO_2_/O_2_ samples compared to packaging in CO_2_/N_2_ and vacuum (*p* < 0.05) (Table 2). Applying an atmosphere containing 67/33% CO_2_/O_2_ was also shown to reduce the production of spoilage metabolites such as hypoxanthine (Hx) and biogenic amines in saithe (*Pollachius virens*) [41]. On day 13, a maximum concentration of 0.60 mmol/100 g of fish muscle of Hx was reached in the inoculated vacuum packaged samples, which was significantly higher than the other groups (*p* < 0.05). There was no difference in the PC level in the inoculated and non-inoculated samples at that time point (*p* = 0.146), whereas the APC level was significantly higher in the inoculated versus non-inoculated samples (*p* < 0.001). Consequently, a potential inoculum effect for the Hx concentration towards the end of storage could not be confirmed.

The concentration of TMA was affected by atmosphere and storage time in the same way as Hx. As seen for the ATP degradation metabolites, storage time and packaging atmosphere had a significant effect on the concentration of TMA (GLM, *p* < 0.001 for both factors), whereas inoculation with *Aeromonas* did not contribute to any differences in the TMA concentration (*p* = 0.352). No TMA was detected in any sample at day 0, but the concentration of TMA increased during storage for all groups except samples with O_2_ in the atmosphere (Table 3). Using O_2_ in the packaging atmosphere is shown to inhibit the formation of TMA as well as the growth of the TMA-producing bacteria in Atlantic cod [27].

Comparable to the studies by Lerfall et al. [42] and Hansen et al. [13], a higher TMA concentration was quantified in the samples packaged in CO_2_/N_2_ and vacuum atmospheres compared to the packages containing CO_2_/O_2_ (*p* < 0.001) at the end of storage. The highest concentrations of TMA were measured in non-inoculated N_2_ samples (5.61 ± 0.36 mmol/100 g) and in VP samples (5.18 ± 0.50 mmol/100 g and 5.30 ± 0.17 mmol/100 g for non-inoculated and inoculated samples, respectively) after 13 days storage. Thus, it is advisable to include O_2_ in the packaging atmosphere of cod to prevent the reduction of TMAO to TMA owing to anaerobic bacterial respiration, which can follow the industrial processing of single vacuum packaged fillets. In the present study, a high concentration of O_2_ was chosen to inhibit the production of TMA. Other studies have indicated that lower concentrations (5–10%) of O_2_ in the packaging atmosphere can reduce the production of TMA [51,52]. Accordingly, Lerfall et al. [42] demonstrated a faster production of TMA in packages completely depleted of O_2_, supporting the importance of oxygen in the atmosphere of TMAO-containing fish species. At the same time, a sufficiently high concentration of CO_2_ is needed to inhibit the growth of other spoilage organisms, e.g., H_2_S-producing bacteria such as *Shewanella* spp. [21,41].

In a previous study, we demonstrated the ability of *A. salmonicida* to grow in inoculated salmon packaged in MA containing 60/40% CO_2_/N_2_ and in a vacuum [30]. In the mentioned study, inoculation of *A. salmonicida* led to a significantly higher concentration of TMA in the VP salmon, whereas the CO_2_-enriched atmosphere reduced the formation of TMA. However, no effect of inoculation with the same strain of *A. salmonicida* on the formation of TMA during storage was detected in the present study, nor on the other metabolites analysed. We hypothesise that the observed difference in the spoilage potential of the same strain in two different marine fish species can be attributed to differences in the chemical and/or microbiological composition of the fish. The TMAO content is normally higher in muscle from Atlantic cod than farmed Atlantic salmon owing to different enzymatic capacity and diet [53]. Hence, the spoilage microbiota of TMAO-rich fish species such as cod is more likely to be dominated by effective TMA-producers such as *P. phosphoreum* [54], which can preclude the interpretation of the actual effect of the inoculated strain in the present study. In salmon, lower in TMAO, it is more likely to find genera of LAB and *Brochothrix thermosphacta* as SSOs [54]. The fact that growth of the inoculated *Aeromonas* strain was more inhibited in cod than salmon in a CO_2_/N_2_ atmosphere might also be related to the higher water content of cod compared to salmon [55], potentially resulting in a better solubility of CO_2_ in the cod muscle and hence an increased effect of the packaging gas.

The present study can serve as a basis for further studies on the spoilage potential of *A. salmonicida* or other *Aeromonas* spp. in packaged, chilled Atlantic cod. It demonstrates that *Aeromonas* can proliferate to high levels (near log 8 CFU/g) in VP cod during refrigerated storage, but we could not detect any effect of the inoculated strain on the production of ATP degradation products or TMA in a naturally contaminated system. Further studies in a more controlled model system such as surface sterilized fish or sterile cod juice might be feasible. Strain-to-strain variation in the spoilage potential of *Aeromonas* is previously reported [8] and might be overcome by applying a mixture of multiple strains. Moreover, a broader analysis of compounds associated with fish spoilage, including acids, alcohol, aldehydes, biogenic amines, and ketones [16,21], is required to assess the role of *A. salmonicida* as a spoilage organism in marine fish species.

## 4. Conclusions

Growth of the inoculated strain *A. salmonicida* in cod was inhibited by applying modified atmospheres consisting of 67% CO_2_ in combination with either 33% N_2_ or O_2_ during storage at 4 °C for 13 days. The combination of vacuum packaging and refrigeration allowed a 3-log increase in the concentration of *Aeromonas* during the storage period. The inoculated strain did not contribute to increased concentrations of Hx or TMA compared to the non-inoculated control in naturally contaminated cod. However, a broader analysis of spoilage metabolites is required to establish the role of *Aeromonas* spp. as a potential spoilage organism in cod. Including O_2_ in the packaging atmosphere inhibited the general bacterial growth more than applying N_2_ as a balancing gas. Moreover, vacuum packaging is not optimal for the shelf-life extension of refrigerated cod as it allows for the rapid growth of TMA- and H_2_S-producing bacteria.

## Figures and Tables

**Table 1:** Experimental groups showing different packaging atmospheres and inoculation status. Inoculation: *Aeromonas salmonicida* strain SU2.

	Packaging Atmosphere
Inoculated	CO_2_/O_2_ (67/33%)
	CO_2_/N_2_ (67/33%)
	Vacuum
Non-inoculated	CO_2_/O_2_ (67/33%)
	CO_2_/N_2_ (67/33%)
	Vacuum

**Table 2:** Average concentrations (mmol/100 g) of inosine monophosphate (IMP), inosine (HxR), and hypoxanthine (Hx) in cod filets with and without inoculation with *A. salmonicida* strain SU2 packaged in CO_2_ (67%) mixed with *either* O_2_ (33%), N_2_ (33%) or packaged in a vacuum. *p*-values for main effect analysis of storage time (P_D_), packaging atmosphere (P_A_), inoculation status (P_I_), and the GLM model (P_M_) are shown in the table.

		Non-Inoculated			Inoculated						
	Day	CO_2_/O_2_ (67/33%)	CO_2_/N_2_ (67/33%)	Vacuum	CO_2_/O_2_ (67/33%)	CO_2_/N_2_ (67/33%)	Vacuum	P_D_	P_A_	P_I_	P_M_
IMP											
	0 *	0.17 ± 0.13 ^a, x^	0.17 ± 0.13 ^a, x^	0.17 ± 0.13 ^a, x^	0.17 ± 0.13 ^a, x^	0.17 ± 0.13 ^a, x^	0.17 ± 0.13 ^a, x^				
	6	0.05 ± 0.0 ^ab, x^	0.0 ± 0.0 ^a, x^	0.0 ± 0.0 ^a, x^	0.08 ± 0.05 ^b, x^	0.01 ± 0.01 ^a, x^	0.0 ± 0.0 ^a, x^				
	13	0.01 ± 0.01 ^a, x^	0.0 ± 0.0 ^a, x^	0.0 ± 0.0 ^a, x^	0.0 ± 0.0 ^a, x^	0.0 ± 0.0 ^a, x^	0.0 ± 0.0 ^a, x^				
	GLM							<0.001	0.734	0.877	<0.001
HxR	0 *	0.37 ± 0.08 ^a, x^	0.37 ± 0.08 ^a, y^	0.37 ± 0.08 ^a, y^	0.37 ± 0.08 ^a, x^	0.37 ± 0.08 ^a, y^	0.37 ± 0.08 ^a, y^				
	6	0.44 ± 0.02 ^b, x^	0.17 ± 0.16 ^ab, xy^	0.21 ± 0.16 ^ab, xy^	0.41 ± 0.01 ^b, x^	0.41 ± 0.05 ^b, y^	0.05 ± 0.02 ^a, y^				
	13	0.37 ± 0.06 ^b, x^	0.01 ± 0.00 ^a, x^	0.0 ± 0.0 ^a, x^	0.36 ± 0.11 ^b, x^	0.01 ± 0.01 ^a, x^	0.0 ± 0.0 ^a, x^				
	GLM							<0.001	<0.001	0.837	<0.001
Hx	0 *	0.04 ± 0.01 ^a, x^	0.04 ± 0.01 ^a, x^	0.04 ± 0.01 ^a, x^	0.04 ± 0.01 ^a, x^	0.04 ± 0.01 ^a, x^	0.04 ± 0.01 ^a, x^				
	6	0.08 ± 0.01 ^a, x^	0.33 ± 0.15 ^ab, y^	0.45 ± 0.24 ^ab, xy^	0.09 ± 0.01 ^ab, y^	0.16 ± 0.05 ^ab, y^	0.49 ± 0.05 ^b, y^				
	13	0.17 ± 0.04 ^a, y^	0.50 ± 0.02 ^b, y^	0.52 ± 0.01 ^b, y^	0.14 ± 0.01 ^a, z^	0.50 ± 0.02 ^b, z^	0.60 ± 0.00 ^c, z^				
	GLM							<0.001	<0.001	0.821	<0.001

* Samples at day 0 are the same for all groups (analysed before packaging). Different lowercase superscript letters within each row (abc) and within each parameter and column (xyz) indicate significant differences (*p* < 0.05) between groups by one-way ANOVA and Tuckey’s pairwise comparison test. *n* = 3 for the samples on day 0, 6 and 13.

**Table 3:** Average concentration (mmol/100 g) of trimethylamine (TMA) in cod filets with and without inoculation with *A. salmonicida* strain SU2 packaged in CO_2_ (67%) mixed with either O_2_ (33%), N_2_ (33%), or packaged in a vacuum. *p*-values for main effect analysis of storage time (P_D_), packaging atmosphere (P_A_), inoculation status (P_I_), and the GLM model (P_M_) are shown in the table.

		Non-Inoculated			Inoculated						
	Day	CO_2_/O_2_ (67/33%)	CO_2_/N_2_ (67/33%)	Vacuum	CO_2_/O_2_ (67/33%)	CO_2_/N_2_ (67/33%)	Vacuum	P_D_	P_A_	P_I_	P_M_
TMA	0 *	0.0 ± 0.0 ^a, x^	0.0 ± 0.0 ^a, x^	0.0 ± 0.0 ^a, x^	0.0 ± 0.0 ^a, x^	0.0 ± 0.0 ^a, x^	0.0 ± 0.0 ^a, x^				
	6	0.0 ± 0.0 ^a, x^	1.58 ± 1.10 ^ab, x^	1.26 ± 0.57 ^ab, x^	0.04 ± 0.04 ^a, x^	0.39 ± 0.19 ^ab, x^	1.84 ± 0.27 ^b, y^				
	13	0.09 ± 0.10 ^a, x^	5.61 ± 0.36 ^c, y^	5.18 ± 0.50 ^c, y^	0.09 ± 0.12 ^a, x^	3.35 ± 0.66 ^b, y^	5.30 ± 0.17 ^c, z^				
	GLM							<0.001	<0.001	0.352	<0.001

* Samples at day 0 are the same for all groups (analysed before packaging). Different lowercase superscript letters within each row (abc) and within each parameter and column (xyz) indicate significant differences (*p* < 0.05) between groups by one-way ANOVA and Tuckey’s pairwise comparison test. *n* = 3 for the samples on day 0, 6 and 13.

## Data Availability

All related data and methods are presented in this paper. Additional inquiries should be addressed to the corresponding author.
